# Cerebral Infarction as a Rare Adverse Event of Immune Checkpoint Inhibitors in Patients With Head and Neck Squamous Cell Carcinoma: A Case Series

**DOI:** 10.7759/cureus.47406

**Published:** 2023-10-20

**Authors:** Takahiro Inoue, Takumi Kumai, Kenzo Ohara, Miki Takahara

**Affiliations:** 1 Department of Otolaryngology, Head and Neck Surgery, Asahikawa Medical University, Asahikawa, JPN

**Keywords:** nivolumab, head and neck squamous cell carcinoma, thromboembolism, cerebral infarction, immune checkpoint inhibitors

## Abstract

Immune checkpoint inhibitors (ICIs) are a novel treatment option for treating head and neck squamous cell carcinoma (HNSCC). Among the immune-related adverse effects, cerebral infarction (CI) is a rare but fatal complication, and it has been reported in various cancers, except HNSCC. Herein, we describe three cases of patients diagnosed with HNSCC who experienced CI following ICI treatment. In addition, we conducted a comprehensive literature review on ICI-related thrombosis. Three patients with recurrent HNSCC were treated with nivolumab. Two patients had a history of CI, or heart disease, and were concurrently prescribed antithrombotic medications during nivolumab treatment. The number of nivolumab administrations varied from 1-25 before the onset of CI. All patients experienced worsening of neurological symptoms due to CI, irrespective of antithrombotic treatment, and they ultimately succumbed to the disease within 16-222 days following their initial ICI administration. ICIs may cause thromboembolisms, leading to CI. Based on our review of the literature, a history of thromboembolism or heart disease could be a risk factor for ICI-related thrombosis.

## Introduction

Immune checkpoint inhibitors (ICIs) have emerged as a novel treatment option for patients diagnosed with head and neck squamous cell carcinomas (HNSCC). The activation of self-reactive immune cells has led to the emergence of multiple immune-related adverse events (irAEs), including dermatological and gastrointestinal effects. Excessive inflammation induces blood coagulation during severe infections. Similarly, nonspecific activation of immune cells by immunotherapy can possibly cause thromboembolism subsequent to blood coagulation. Supporting this hypothesis, several case reports have shown that the incidence rate of venous and arterial thromboembolic events with ICIs is 2.7%-12.9% and 1.1%-1.8%, respectively [1-2], which is comparable to that with cisplatin [3]. Thus far, thromboembolisms following administration of ICIs in patients diagnosed with HNSCC have not been reported. Herein, we report three cases of patients diagnosed with HNSCC who experienced cerebral infarction (CI) after treatment with ICIs.

## Case presentation

Case 1

A 67-year-old man initially presenting with a sore throat was referred to our hospital. He had a history of hypertension, diabetes, CI, and treatment for esophageal cancer with combined chemoradiotherapy (CCRT) received five years earlier. He had been receiving clopidogrel to prevent CI. Computed tomography (CT), magnetic resonance imaging (MRI), positron emission tomography (PET), and laryngeal fiberscopy revealed the presence of an oropharyngeal tumor. Following a tumor biopsy, the patient was diagnosed with clinical stage IVa (T4aN0M0) right oropharyngeal cancer.

**Figure 1:**
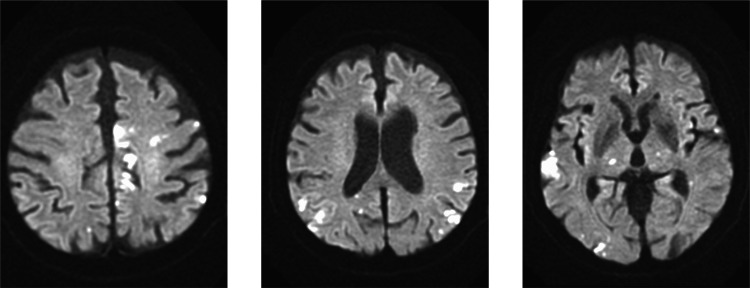
Findings of magnetic resonance imaging (MRI) in case 1 MRI scan reveals multiple cerebral infarctions (diffusion-weighted MRI scan), which developed four days after the second nivolumab administration.

The patient was treated with cisplatin-based chemoradiotherapy. A PET scan acquired two months post-treatment revealed right neck lymph node metastasis; consequently, TS-1 was prescribed for 27 months. After 34 months of the initial treatment, left cervical and mediastinal lymph node metastases were observed. The patient underwent a regimen involving cisplatin, fluorouracil, and cetuximab, followed by paclitaxel and cetuximab for 19 weeks. Because the mediastinal lymph nodes were enlarged despite chemotherapy, the patient was initiated on nivolumab treatment. After receiving two courses of nivolumab over four days, the patient started experiencing numbness of the mouth and arms. An MRI scan revealed multiple new CIs (Figure 1).

Despite receiving heparin and edaravone, his paralysis worsened, leading to the development of right hemiplegia and aphasia. The patient succumbed to CI at 114 days after nivolumab administration.

Case 2

A 75-year-old man with a history of aortic valve regurgitation and atrial fibrillation, managed with catheter ablation and rivaroxaban, sought medical attention for a sore throat and shortness of breath at a private clinic. Subsequently, he was referred to our department for treatment of a tumor in the right pyriform sinus. Based on the biopsy and radiological findings, he was diagnosed with right hypopharyngeal cancer of clinical stage Ⅳb (T4aN3bM0). The patient was treated with CCRT using docetaxel and cisplatin. He developed metastases to the liver and vertebrae during CCRT, necessitating discontinuation of the treatment, and received palliative radiotherapy to address the vertebrae-related issues.

**Figure 2:**
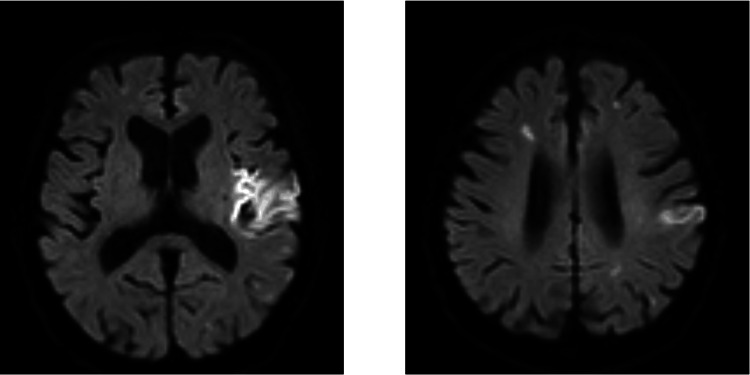
Findings of magnetic resonance imaging (MRI) in case 2 MRI scan reveals multiple cerebral infarctions (diffusion-weighted MRI scan), which developed six days after the first nivolumab administration.

Following palliative radiotherapy, the patient was initiated on nivolumab. Consequently, he developed aphasia and right hemiplegia and was diagnosed with CI, as revealed through a brain MRI conducted six days after nivolumab administration (Figure 2).

Following consultation with neurologists, the patient was initiated on edaravone. Despite these interventions, his general condition gradually worsened, and he succumbed to death after 16 days of nivolumab administration.

Case 3

A 62-year-old man visited a private clinic to seek medical attention for a headache. An MRI revealed a mass in the left maxillary sinus. Subsequently, he was referred to our department, where he was diagnosed with left maxillary sinus cancer of clinical stage Ⅳb (T4bN2bM0). The patient was treated with CCRT using docetaxel and cisplatin. However, due to the persistent local tumor, three courses of chemotherapy using cetuximab, cisplatin, and fluorouracil were administered. Following chemotherapy, the patient received 25 courses of nivolumab, resulting in the complete remission of the tumor. The patient developed disturbances in consciousness and weakness of the right upper and lower limbs 31 days after the last nivolumab administration. The patient was diagnosed with CI, and he succumbed to death 222 days after the initial nivolumab administration.

## Discussion

The irAEs typically involve the skin, liver, renal system, and endocrine system. Although confirming thromboembolism as an irAE can be challenging, in this report, we have described three cases of CI following PD-1 blockade therapy. Recently, thrombosis associated with ICIs has been regarded as ICI-associated thrombosis (IAT) [4]. Kunimasa et al. reported a case of pulmonary embolism following pembrolizumab treatment for a patient with lung cancer [5]. Horio et al. reported a case of Trousseau’s syndrome triggered by pembrolizumab in a patient with lung cancer [6]. Meanwhile, Cautela et al. reported a case of acute coronary syndrome associated with nivolumab in a patient with lung cancer [7]. These cases suggest that ICIs could induce thromboembolism in patients with HNSCC.

Off-target immune responses affecting the vascular endothelium could serve as a trigger for vasculitis during ICI treatment. Vasculitis initiated by immune cells may serve as a risk factor for thrombosis due to the activation of coagulation factors and endothelial impairment [8]. Tissue factors, which activate factor VII and initiate the extrinsic pathway of thrombogenesis [9], are possibly released from cancer cells damaged by immune cells, thus initiating coagulation. Among thromboembolic events, CI is particularly fatal and requires prompt diagnosis. To date, 21 cases of CI following ICI treatment have been reported, including ours (Table 1) [7,10-20].

**Table 1:** Summary of reported cases of cerebral infarction following treatment with immune checkpoint inhibitors. *N/A: Not applicable.

Case	Year	Author	Age/Sex	Cancer type	TNM	Stage	ICIs	Line	ICIs Course (times)	Days from first administration of ICIs to onset of CI (days）	Outcome
1	2018	Yoneda, et al [10].	77/F	Lung SCC	T4N2M1c	ⅣB	Nivolumab	2	1	24	died
2	2018	Horio, et al [7].	66/M	Lung Adenocarcinoma	T1cN3M1c	ⅣB	Pembrolizumab	1	1	4	died
3	2018	Tsukamoto, et al [11].	56/M	Lung Adenocarcinoma	N/A	N/A	Pembrolizumab	1	3	21	PR
4	2019	Hasegawa, et al [12].	82/F	Lung Adenocarcinoma	T3N0M0	IIB	Nivolumab	2	2	36	died
5	2019	Nakao, et al [13].	63/F	Lung Adenocarcinoma	T2aN3M1b	ⅣA	Pembrolizumab	2	1	11	PR
6	2019	Sato, et al [14].	72/M	Lung SCC	N/A	ⅢA	Atezolizumab	3	1	8	N/A
7	2019	Sato, et al [14].	71/M	Lung Adenocarcinoma	N/A	ⅣB	Pembrolizumab	1	1	N/A	CR
8	2019	Sato, et al [14].	67/M	Lung SCC	N/A	ⅣB	Pembrolizumab	1	12	N/A	PR
9	2020	Nishimura, et al [15].	66F	Lung Adenocarcinoma	T2aN3M0	ⅢB	Atezolizumab	2	2	52	PR
10	2020	Ando, et al [20].	78/-	Kidney Cancer	N/A	N/A	Nivolumab	4	N/A	178	N/A
11	2020	Ando, et al [20].	57--	Esophageal Cancer	N/A	N/A	Nivolumab	4	N/A	54	N/A
12	2020	Ando, et al [20].	74/-	Stomach Cancer	N/A	N/A	Nivolumab	3	N/A	6	N/A
13	2020	Ando, et al [20].	78/-	Lung Cancer	N/A	N/A	Pembrolizumab	1	N/A	37	N/A
14	2020	Ando, et al [20].	79/-	Lung Cancer	N/A	N/A	Pembrolizumab	3	N/A	110	N/A
15	2021	Shinoya, et al [16].	68/M	Lung Adenocarcinoma	T4N2M0	ⅢB	Pembrolizumab	2	N/A	35	died
16	2021	Fu, et al [17].	70/M	Metastatic Cholangiocarcinoma	N/A	N/A	Pembrolizumab	1	3	60	died
17	2022	Tokuda, et al [18].	70/M	Lung Cancer	N/A	ⅠB	Nivolumab	2	N/A	60	CR
18	2022	Zao, et al [19].	48/F	Lung Adenocarcinoma	T1bN3M1a	ⅣA	Pembrolizumab	1	1	4	died
19	2023	Present Case 1	67/M	Oropharyngeal Cancer	T4aN0M0	ⅣA	Nivolumab	4	2	4	died
20	2023	Present Case 2	75/M	Hypopharyngeal Cancer	T4aN3bM0	ⅣB	Nivolumab	2	1	6	died
21	2023	Present Case 3	62/M	Maxillary Sinus Cancer	T4bN2bM0	ⅣB	Nivolumab	3	25	222	died

**Figure 3:**
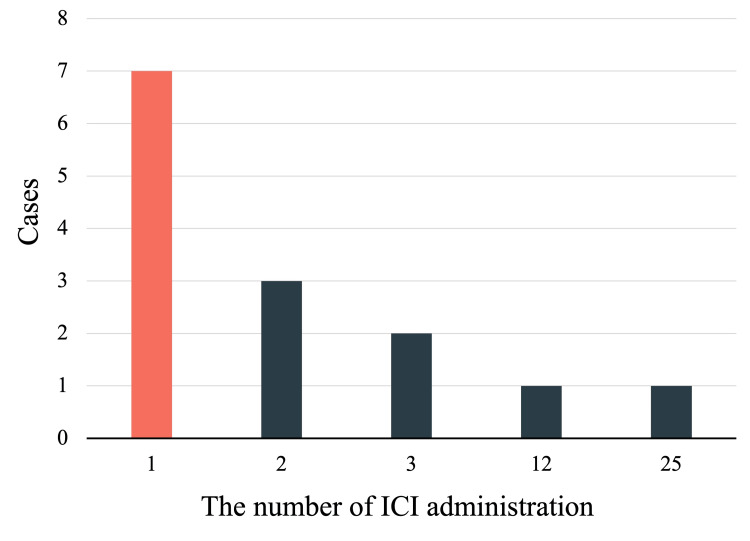
Administration of doses of immune checkpoint inhibitors (ICIs) until the onset of cerebral infarction Summary of ICI doses in reported cases, including ours. The x-axis depicts the number of ICI treatments until the onset of cerebral infarction, and the y-axis depicts the number of reported cases.

Among the included patients, 11 were men, and four were women (others were not specified). The median age of the patients was 70 (range: 48-82) years. Fourteen patients had lung cancer, and three patients had HNSCC (present cases). The most commonly used ICI was pembrolizumab in 10 cases, nivolumab in nine cases, and atezolizumab in two cases. Most patients developed CI after the first ICI administration (Figure 3).

Therefore, attention to neurological symptoms is warranted, especially after the initial administration of ICIs. The median time from the first ICI administration to the onset of CI was 35 (range: 4-222) days. The risk factors for cancer-associated thrombosis in patients treated with ICIs are considered to include a history of thromboembolism and heart disease, as well as the use of antiplatelet and anticoagulant therapy. In our study, Case 1 was administered clopidogrel for managing CI, and Case 2 was prescribed rivaroxaban for managing atrial fibrillation. The risk of thromboembolism during ICIs treatment should be considered in patients with a history of such conditions or prescriptions.

## Conclusions

We reported three cases of patients with HNSCC who developed CI following ICI treatment. Thromboembolic events, including CI, are rare but fatal irAEs. A prompt MRI examination is necessary to diagnose IAT when a patient develops neurological symptoms during ICI treatment.
